# Mitral Stenosis and Regurgitation due to Late Prosthetic Aortic Valve Migration

**DOI:** 10.1016/j.atssr.2026.02.011

**Published:** 2026-02-25

**Authors:** Taro Nakazato, Daisuke Yoshioka, Tatsuya Ozaki, Mutsunori Kitahara

**Affiliations:** 1 Department of Cardiovascular Surgery, Kansai Rosai Hospital, Amagasaki, Japan; 2 Division of Cardiovascular Surgery, Department of Surgery, Osaka University Graduate School of Medicine, Suita, Japan

## Abstract

We report a rare case of mitral stenosis and regurgitation after aortic valve replacement due to prosthetic valve migration. A 74-year-old woman underwent aortic valve replacement using a Carpentier-Edwards PERIMOUNT valve (Edwards Lifesciences) 9 years prior. Six years postoperatively, outpatient echocardiography incidentally revealed abnormal rocking motion of the bioprosthesis, with no signs of infection or paravalvular leak. Finally, the bioprosthetic valve interfered with the anterior mitral valve leaflet, causing severe mitral stenosis and regurgitation. Redo aortic valve replacement and mitral valve replacement with the commando procedure were performed, and pathologic findings revealed no evidence of connective tissue disease, aortitis, or infection.

Late prosthetic valve migration is a critical and rare complication after sutureless aortic valve replacement (AVR) or transcatheter aortic valve replacement (TAVR).^1^, ^2^, ^3^ It scarcely occurs after conventional AVR sewn to the annulus. Herein, we report a rare surgical case of late prosthetic valve migration without a paravalvular leak that caused mitral stenosis and regurgitation.

A 74-year-old woman underwent AVR with a 23-mm Carpentier-Edwards PERIMOUNT valve (Edwards Lifesciences) and coronary artery bypass grafting for aortic regurgitation and coronary artery disease 9 years prior. The aortic valve was tricuspid, there was no obvious difference in the size of the cusps, and the cause of the aortic regurgitation was due to annular dilatation. AVR was performed with a standard technique including the use of noneverting pledgeted sutures and postoperative echocardiography showed no obvious abnormalities (Figure 1A, Supplemental Video 1). Six years later, outpatient echocardiography revealed that the bioprosthesis exhibited an abnormal rocking motion and slightly migrated towards the left ventricle (LV); no paravalvular leak was observed. The patient was afebrile at the time, and laboratory investigations exhibited no inflammatory changes. Subsequently, an outpatient echocardiography revealed that the bioprosthesis gradually migrated to the LV, still without a paravalvular leak, but eventually interfered with the anterior mitral valve leaflet, causing severe mitral stenosis and regurgitation (Figures 1B, 1C, Supplemental Video 2). Electrocardiogram-synchronized enhanced computed tomography (CT) showed the migration of the bioprosthesis into the LV compared to the preoperative CT images of the initial surgery (Figure 1D, Supplemental Figure 1). Positron emission CT demonstrated no fluorodeoxyglucose uptake in the aorta, and additional blood tests ruled out autoimmune disorders. Redo AVR and mitral valve replacement was planned because of worsening dyspnea on exertion and elevated brain natriuretic peptide levels. The complete surgical details are presented in Supplemental Video 3. After successful resternotomy without injury, cardiopulmonary bypass was performed by cannulation of the ascending aorta, superior vena cava, and inferior vena cava. After clamping the aorta, aortotomy was performed, and the bioprosthesis was confirmed to have migrated to the LV and adhered to the surrounding tissue (Figures 2A, 2B). The bioprosthesis was easily removed using a harmonic scalpel; however, the aortomitral curtain and anterior mitral valve leaflet had degenerated owing to long-term prosthetic valve interference. Redo AVR (25-mm bioprosthesis) and mitral valve replacement (27-mm bioprosthesis) were performed using the Commando procedure. To prevent prosthetic valve migration, horizontal mattress sutures were placed along a new aortomitral curtain made of a bovine pericardial patch. Furthermore, the aortic bioprosthetic valve was fixed more securely by placing horizontal mattress sutures from the right ventricle or right atrium. The pathologic findings of the aortic wall showed no evidence of connective tissue disease, aortitis, or infection (Figure 2C). The patient was transferred to a different hospital for rehabilitation on postoperative day 79 and is currently undergoing follow-up. Postoperative echocardiography revealed no paravalvular leak or abnormal rocking motion of the bioprosthetic valves (Supplemental Video 4).

**Figure 1:**
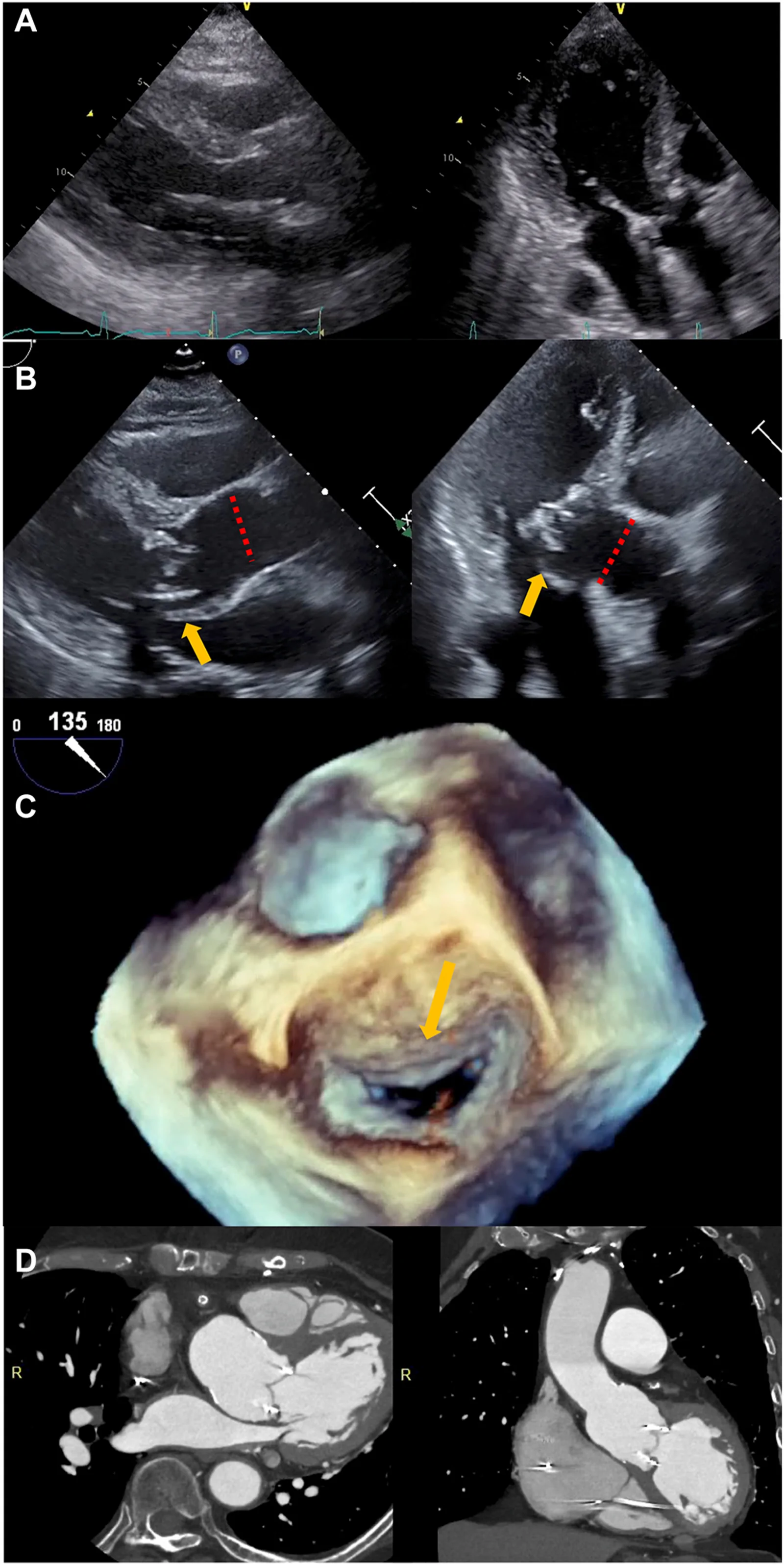
(A) Transthoracic echocardiography 1 week after the first surgery shows no obvious abnormalities from the left ventricular long axis view (left side) and apical long axis view (right side). (B) Transthoracic echocardiography 9 years after the first surgery shows the migrated bioprosthesis (red dotted line: the aortic annulus) interferes with the anterior mitral leaflet (the yellow arrows) from the left ventricular long axis view (left side) and apical long axis view (right side). (C) Three-dimensional transesophageal echocardiography shows restricted opening of the anterior mitral leaflet due to the bioprosthesis’ interference (yellow arrow). (D) Electrocardiogram-synchronized enhanced computed tomography shows the migration of the bioprosthesis into the left ventricle from the 3-chamber (left side) and 4-chamber view (right side).

**Figure 2:**
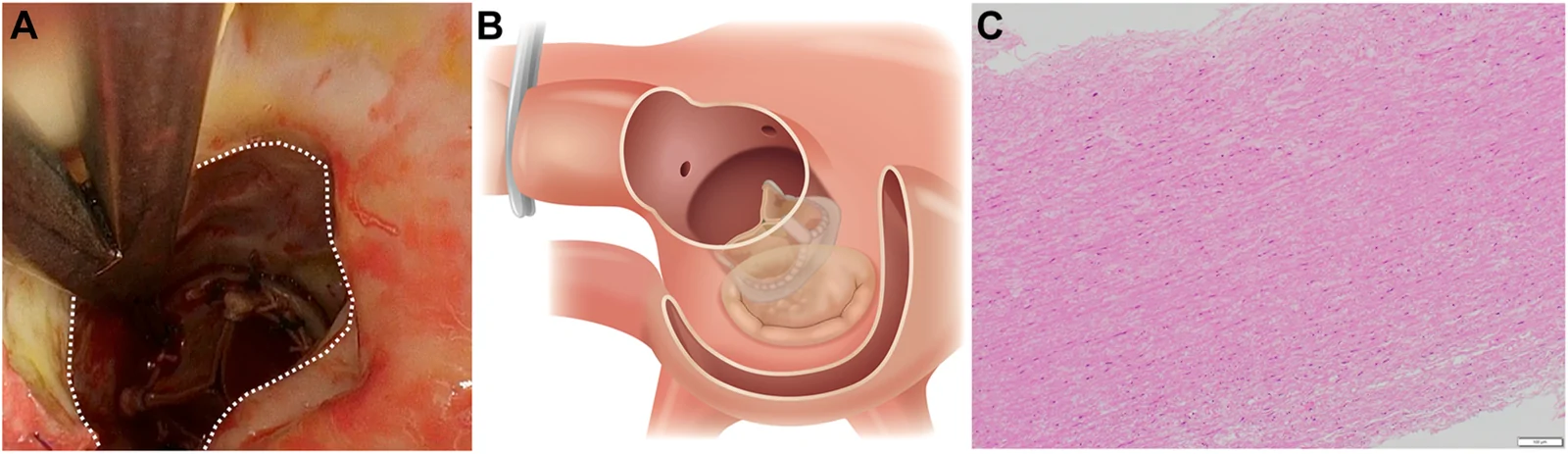
(A) Intraoperative endoscopy shows the bioprosthesis that has migrated from the aortic annulus (white dotted line) to the left ventricle. (B) Schema showing the positional relationship of the bioprosthesis and mitral valve. (C) Hematoxylin-eosin staining showing almost no inflammatory changes within the aortic wall.

## Comment

Prosthetic valve migration is often associated with paravalvular leaks, potentially leading to heart failure. This case is extremely rare because the valve migration occurred after AVR with interrupted pledgeted suture technique rather than after sutureless AVR or TAVR, and no paravalvular leak was observed. A similar case has been reported previously, in which the aortic root was enlarged vertically, presented with a valve detachment–like pathology due to Takayasu arteritis.^4^ The differences between this report and the present case are whether the prosthetic valve had been detached from the aortic annulus and whether the patient had an underlying disease such as aortitis. In this case, various tests indicated no obvious connective tissue disease, aortitis, or autoimmune disorders. As the bioprosthesis interfered with the anterior mitral leaflet, its detachment from the annulus was confirmed. The cause of prosthetic valve migration might be technical error at the initial operation, such as the sutures being not in the annulus. However, postoperative transthoracic esophagectomy (Figure 1) confirmed the bioprosthesis was properly placed. If the sutures had not been in the annulus, it is likely that the bioprosthesis would have migrated soon after the initial surgery (before 6 years had passed). Furthermore, since the entire bioprosthesis migrated, rather than just a part of it, it is difficult to believe that most of the sutures were not in the annulus. Based on the this, it was thought that there were no technical errors in the initial operation. The CT before the initial operation showed that the perimeter-derived diameter of the aortic annulus was 25 mm, which ruled out the possibility that an oversized or undersized bioprosthesis had been placed. The cause of migration in our case is unclear, but it is reasonable to speculate that there was local inflammation (in the annulus), which caused the valve to detach. Regarding reoperation for valve migration, in cases after TAVR, redo-TAVR may be a relatively safe and effective option.^3^ Contrarily, in cases after sutureless AVR or conventional AVR, resternotomy is often required. In some cases, the bioprosthesis can be easily removed, however, other cases might require double valve replacement or aortic root surgery, which can be difficult.^1^^,^^2^^,^^4^ In this study, the Commando procedure for double-valve replacement was selected because the aortomitral curtain had degenerated, the aortic bioprosthesis was required to be fixed to the bovine pericardial patch to prevent migration, and a good surgical field of view was important.^5^ Transection of the superior vena cava provided a better surgical field of view and facilitated double-valve replacement. As the pathology of valve migration is unknown, strict lifelong follow-up is required.

## Figures and Tables

**Supplemental Figure 1:**
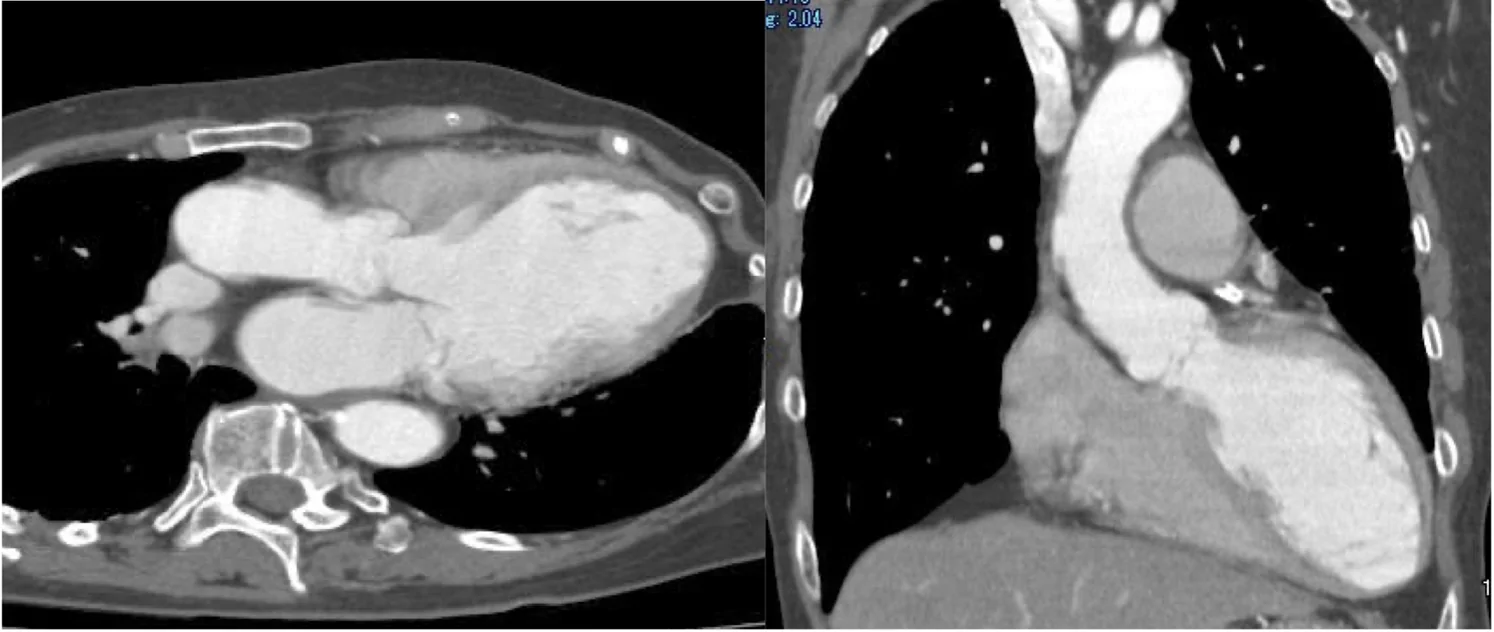


## References

[1] Amr G., Ghoneim A., Giraldeau G., Demers P. (2017). First case of a sutureless Perceval valve delayed proximal migration. J Thorac Cardiovasc Surg.

[2] Wacker M., Slottosch I., Scherner M., Varghese S., Wippermann J. (2019). Late onset valve dislocation of the Edwards Intuity rapid-deployment bioprosthesis. Ann Thorac Surg.

[3] Lauten A., Hamadanchi A., Doenst T., Figulla H.R. (2013). Late migration of balloon-expandable transcatheter aortic valve. Eur Heart J.

[4] Kiryu K., Igarashi I., Wada T., Yamamoto H. (2022). Subvalvular tissue mimicking valve detachment-like pathology by vertical aneurysm in Takayasu's arteritis. Interact Cardiovasc Thorac Surg.

[5] David T.E., Lafreniere-Roula M., David C.M., Issa H. (2022). Outcomes of combined aortic and mitral valve replacement with reconstruction of the fibrous skeleton of the heart. J Thorac Cardiovasc Surg.

